# Two Cases of Spina Bifida—One of them Cured

**Published:** 1847-01

**Authors:** 


*Two cases of Spina Bifida—one of them Cured.*—Mr. Dumville exhibited a preparation of spina bifida taken from a child aged five months. The arrest of developement happened in the spinous processes of the third, fourth, and fifth lumbar, and first and second sacral vertebræ, which gaped widely apart. The sac in which the cauda equina partly expanded was large enough to contain an ounce of fluid. The tumour was punctured at the urgent instance of the friends, but at a period when the failing powers of life offered scarcely a hope of recovery. The child died with the usual symptoms.

Mr. Dumville related the particulars of another case, in which the congenital deficiency was situate in the same locality. He evacuated the sac by puncture, and the recovery was complete. The child, which was nine months old at the time of the operation, has now attained eighteen months; and its health is unexceptionable. The operation was quickly followed, in this instance, by a profuse perspiration; the face immediately became deeply congested; and there was much derangement of the stomach, attended with vomiting. The child was very fretful, had excessive twitchings, but no distinct convulsion. When the sac was quite empty all the symptoms were aggravated; but on becoming again distended with secretion, which occasionally took place in consequence of mechanical obstruction of the aperture, the symptoms underwent amelioration. The discharge of fluid, with more or less of the symptoms described, having lasted fourteen days, the orifice then healed over, and no further secretion of serum following, the child convalesced without any relapse. This case he adduced rather as an exception to, than as an example of a general rule.

Judging from the aggravation of the symptoms whenever any excess of the fluid happened to drain off, it would appear that too much precaution cannot be taken in order to secure the evacuation of the sac as gradually and in as constant a manner as possible, in these cases, and that the plan of operation most suitable for the attainment of this desirable end should be adopted, and, above all, timely put in practice.—*Ibid.*

